# Elliptically polarized high-harmonic radiation for production of isolated attosecond pulses

**DOI:** 10.1038/s41598-021-88557-1

**Published:** 2021-05-05

**Authors:** Ulrich Bengs, Nickolai Zhavoronkov

**Affiliations:** Max-Born Institut Berlin, Max-Born Strasse 2A, 12489 Berlin, Germany

**Keywords:** Optics and photonics, Optical techniques

## Abstract

Circularly polarized attosecond pulses are powerful tool to study chiral light-matter interaction via chiral electron dynamics. However, access to isolated circularly polarized attosecond pulses enabling straightforward interpretation of measurements, still remains a challenge. In this work, we experimentally demonstrate the generation of highly elliptically polarized high-harmonics in a two-color, bi-circular, collinear laser field. The intensity and shape of the combined few-cycle driving radiation is optimized to produce a broadband continuum with enhanced spectral chirality in the range of 15-55 eV supporting the generation of isolated attosecond pulses with duration down to 150 as. We apply spectrally resolved polarimetry to determine the full Stokes vector of different spectral components of the continuum, yielding a homogenous helicity distribution with ellipticity in the range of 0.8-0.95 and a negligible unpolarized content.

## Introduction

High-harmonic generation (HHG) is a strong field process driven by the nonlinear interaction of laser radiation with different gaseous as well as condensed media. During this interaction a laser field is upconverted through ionization-acceleration-recollision events to coherent radiation of very short wavelengths with duration down to a few tens of attoseconds (1 as=$10^{-18}$s)^1–3^. Many frontiers of attosecond science, which studies the behavior of electrons in atoms and molecules rely on HHG technology not only as a source of radiation with unique properties^4–7^, but also as an analytic tool for high-harmonic spectroscopy^8,9^.

Controlling amplitude, frequency and polarization of the generated XUV radiation is essential to unlock the true potential of HHG as a source for advanced experiments. In particular polarization, as a fundamental property of light, determining the way of light-matter interaction, attracts strong motivation for extending the accessible polarization states in HHG to enable attosecond-resolved measurements of chiral-sensitive electron dynamics. In principle, synchrotron and free-electron laser sources with tunable polarization ^10–13^ provide the brightness and cover the spectral range for such measurements, yet pulse durations in the attosecond region are unavailable. Additionally, as large-scale experiments the availability is limited making a table-top source highly attractive.

The constricted access to a variety of polarization states originates fundamentally out of the recollision nature of generation: the high harmonic polarization follows the atomic dipole direction, directly related to the driving laser beam polarization, however, even moderate ellipticity drastically decreases the recombination probability and the efficiency of harmonic generation^14^.

Alternatively, the polarization of high harmonics generated by a linearly polarized laser can be converted towards elliptical one using a reflective phase retarder^7,15^. Although the robustness and seeming simplicity of this approach still attract numerous groups to contribute to this topic^16^ it has serious drawbacks in form of a technically challenging realization, high losses and narrow bandwidth.

Other, more sophisticated schemes have been proposed to extend harmonic polarization towards circular by focusing on harmonic generation in aligned molecular assemblies by directly application of linearly or slightly elliptically polarized laser field^9,17–20^. The resulting radiation is however not entirely elliptically polarized while the maximum ellipticity stays well below $\varepsilon$ = 0.45.There are also another theoretical approaches predicting the emission of isolated attosecond pulses originating from HHG in different aligned molecules^21,22^, which yet have to prove their experimental feasibility.

There is another type of approaches based on application of tailored driving laser fields^23–25^ that have proven to be feasible over a period of development and tests: two-colour bi-circular (TCBC) collinear schema introduced by a pioneering experimental work^26^, and later proposed noncollinear geometry with one or two-colour laser driving radiation^27^.

In the noncollinear scheme two circularly polarized femtosecond beams of equal-intensity, but opposite helicity are focused at an angle of a few degrees in a gas target, producing at the each local position within the crossing area a linearly polarized field with the polarization rotating as a sinusoidal function of the transverse position^27^. This causes a transverse modulation of the atomic dipole moments in the interaction region demonstrating as a result circularly polarized odd harmonics in the far-field. The harmonics are angularly well separated by helicity and from the driving beams. Undeniably it is in benefit for many applications to have separated pump and harmonics beams, but also gives rise to a an inherent nonlinear spatial chirp and undesirable spatio-temporal distortion and limitation by a short interaction length and transverse-walk-off^28,29^. Polarization control of high-harmonics supporting generation of isolated pulses has recently been realized by utilizing the noncollinear approach^30^.

The two-colour bi-circular (TCBC) approach also offers full polarization control of generated harmonics without decreasing the upconversion efficiency^31–33^. The harmonics are driven by two colour collinear coherent laser beams, both circularly polarized, but with the opposite helicities. The combined laser field has a folded three dimensional rosette shape with some maxima per fundamental period. Strong field ionization experiment illustrates this particular field shape^34^. The exact spectral position and separation of the harmonic orders depend on the spectral content of the driving radiation^35^. However, although the generated high harmonics are circularly polarized with $|\varepsilon |\approx$1 the same does not necessarily apply to the generated attosecond pulses. The reason is that pairs of adjacent harmonics are counter-rotating and the overall chirality cancels since the pairs of counter-rotating harmonics have a similar intensity. This particularity poses a major obstacle in the generation of spatial chirp free isolated attosecond pulses with pure elliptical polarization. Recent studies however show the possibilities to select harmonics of one helicity, resulting in increasing of spectral chirality and in overall elliptically polarized pulses. On the mircoscopic level, the contrast between neighbouring harmonics of opposite helicity has been shown to increase in Neon and is sensitive to the intensity ratio between the driving fields ^32,36^) yielding attosecond pulse trains with ellipticity up to 0.77 ^37^. Moreover, the opimization of phase-matching conditions^33,38^ proves further to enhance harmonics of one helicity. The combination of these optimal elements creates a “helicity dependent filter” paving the way for the TCBC approach to become a promising candidate to produce highly elliptically polarized pulse trains as well as isolated pulses^32,39^.

Here we experimentally utilize for a first time the two-colour bi-circular approach with few-cycle driving laser pulses to generate a XUV-continuum spanning up to 55 eV promising to support highly elliptical isolated attosecond pulses. Implementation of helicity dependent filter allows us to support generation of the XUV-radiation having the helicity of the fundamental driving beam. We apply a modified version of rotating-analyzer ellipsometry with compensator (RAEC) to determine ellipticity, helicity and degree of polarisation for different parts of the generated spectrum. The radiation with the spectral contain of the characterized parts is approaching the ellipticity of $\varepsilon$ $\ge$0.85 with only 5% of unpolarized part and supports isolated attosecond pulses with duration as short as 190 as, while the whole continuum corresponds to a fourier-limited pulse as short as 150 as.

## Generation of chiral XUV continuum

**Figure 1:**
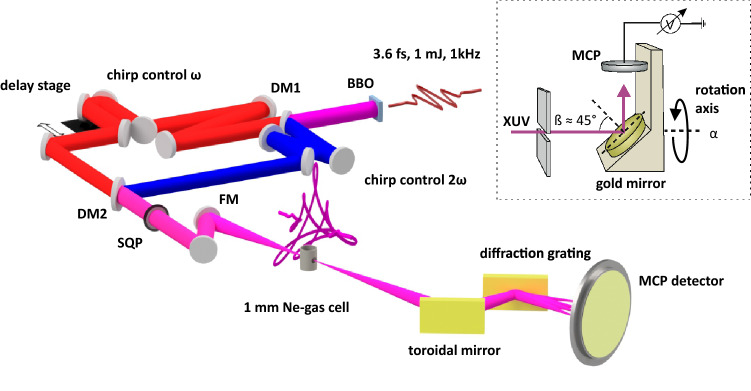
Experimental setup for the generation of circularly polarized harmonics in TCBC approach. The elements: BBO- beta-barium borate crystal; DM1, DM2- dichroic mirrors; SQP- superachromatic quater-wave-plate; FM- focusing mirror f = 350 mm; MCP- detector for XUV radiation. The analyer which replaces the MCP-detector for polarization measurements is shown in the insert, where $\beta$ is the incidence angle and $\alpha$ is the rotation (azimuthal) angle of the analyzer.

We have used few-cycle pulses originating from a Ti:sapphire-based laser system with a single stage regenerative amplifier followed by pulse compression in a hollow-core-fiber in order to realize the TCBC approach. Pulses with 4.8 fs duration for the fundamental radiation and 7.5 fs for the second harmonic were combined collinearly in one beam and focused to the intensities of $I(w)\approx 3.5\times 10^{14}\,W/cm^{2}$ and $I(2w)\approx 5\times 10^{13}\,W/cm^2$ in 0.9 mm thick Ne-cell target (see “Methods” and Supplementary C for more details). The polarizations of driving field components were determined to be clockwise circular for the fundamental $\epsilon _{\omega }\approx 0.95\pm 0.02$ and counter-rotating for the second harmonic $\epsilon _{2\omega }\approx 0.88\pm 0.02$ (minor to major axis ratio of the polarization ellipse with major axis along s-polarization). The entire setup is depicted in Fig. 1.

**Figure 2:**
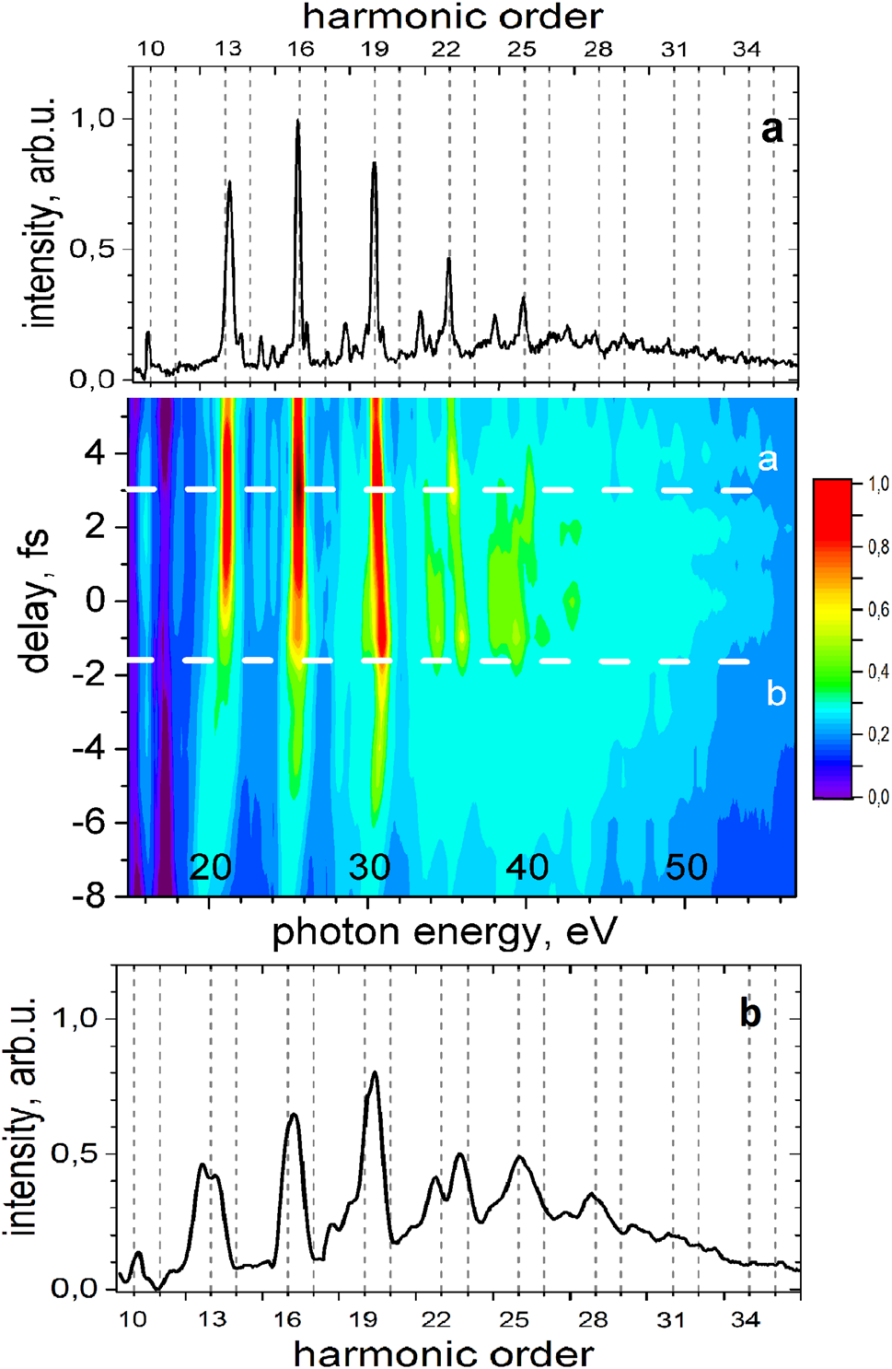
Spectrum of high harmonics generated in two-colour bi-circular approach with few-cycle pulses. The 2D-color figure present the spectra for different time delays between fundamental and second harmonic driving fields, the upper **(a)** and lower **(b)** panels present the harmonics spectra for time delays +3 fs and −1.8 fs.

According to the selection rules the generated XUV-radiation consists of circularly polarized harmonics of orders $3n+1$ having the helicity of the fundamental beam and orders $3n+2$ following the helicity of the second harmonic beam, while the 3*n* harmonics are forbidden^23^(see Supplementary A for more details). To generate a highly elliptically polarized XUV spectrum we applied the helicity dependent filter for harmonics generated in neon^39,40^ implemented and confirmed earlier as a combination of optimized phase-matching^33,38^ and an increased intensity ratio between fundamental and second harmonic^32,35–37^. Additionally, we tune the temporal overlap between the two-colour components to adjust conditions for generation of isolated pulses^39^. The generation of XUV-radiation shown in Fig. 2 was observed only within the temporal window where the two driving pules overlap. The shape of the XUV-spectrum manifests a strong asymmetry in shape regarding zero delay on the timing between the driving pulses. For positive delays (fundamental pulse precedes) the spectrum consists of narrow harmonics, whose bandwidth increases up to five times while going towards negative delays.

The asymmetry and changes in the spectral bandwidth can be qualitatively related to the asymmetrical shape of the fundamental 4.8 fs pulse having a trailing satellite of about 15% intensity as well as a weak 20 fs long background (see Supplementary C for more details). For positive delays the $\sim$ 8 fs second harmonic pulse overlaps with both the fundamental main pulse and its satellite, where the intensity ratio *I*(*w*)/*I*(2*w*) can be also different, thereby numerous generation events interfere to narrower harmonics and stronger modulated features in the spectrum. For negative delays the interaction window is constrained between the falling edge of the second harmonic pulse and the steep rising edge of the fundamental pulse, resulting in generation of only a few attosecond flashes and therefore much broader harmonics and a continuum in the cut-off region (see Fig. 2). The low order harmonics 13, 16, 19 are spectrally broad demonstrating a very high contrast to the barely discernable harmonics of orders $3n+2$ and 3*n*.The harmonics above order 22 show an increasing spectral bandwidth exceeding an IR-photon’s energy and merge to a continuum in the cut-off region. The important feature inherent to alls delays is the concentration of the radiation in the vicinity of $3n+1$ harmonics over other orders.

To verify the ability of the TCBC approach to generate highly elliptical attosecond pulses, a key element that needs to be determined is the exact polarization of the XUV radiation, the challenging problem, which we will address in the next section. Such measurements present also a good basis to prove the conclusions of the modeling ^32,39^ supporting generation of isolated attosecond pulses with high ellipticity in TCBC approach.

## Determination of the polarization state

According to symmetry considerations in the TCBC approach the harmonics are circularly polarized with ellipticities $\varepsilon$ = ±1. This conclusion is however only valid for driving pulses of few tens of femtosecond, with the fields counter-rotating exactly in the same plane and perfect circular polarization where the produced harmonics are narrow. However, in any realistic experiment there will be some deviation from this ideal case. In^41,42^ the implications of having imperfect circular polarization for the second harmonic beam such that $\varepsilon$(2$\omega$) < 0.9 on harmonics ellipticity was considered, with the result, that the absolute value of the harmonic ellipticity generally increases with increasing harmonic order and influence of this imperfection is much larger on the ellipticity of $3n+2$ harmonics than on $3n+1$ harmonics, providing $\varepsilon$(H13) > 0.9, whereas $\varepsilon$(H11) < 0.62. Another issue to address is the breaking of symmetry between the three atto-pulses within the $\omega$ cycle that result in a decreased harmonic ellipticity manifesting in the emission of the 3n harmonics and possibly depolarization^42^. Such breaking can be induced either by the elliptical polarization of the driving fields^41,43^, imperfect temporal overlap^23^ or by the anisotropic generating medium ^36^.

Some doubts also were placed based on simulations and measurements of the polarization state for some individual harmonics by the method of molecular polarimetry^42^. The origin of the depolarization was figured out to be a considerable broadening of the harmonic peaks by application of few-cycle driving pulses or by increasing harmonic orders that leads to overlap of neighboring harmonics with opposite helicity and mixing of their polarization properties^42^. It is obvious that this issue is less relevant for the approach followed here due to the evidently high contrast between parts of the XUV spectra with different helicity. The exact knowledge about polarization of each contributing spectral parts is required to determine a chirality the spectrum and to estimate the polarization state of attosecond pulses. The tomographic reconstruction of high harmonics^44^ and the molecular frame photoelectron angular distributions in molecular dissociative photoionization^45^ was recently applied to probe the polarization state of circularly polarized individual harmonics. Although these methods are extremely challenging in practical realization requiring very sophisticated experimental equipment, materials and data analyzing procedures they do not deliver conclusive information about the temporal structure (see Supplementary B for more details).

In this work, we determine the polarization state of the generated XUV-continuum by utilizing a polarizer setup based on a single Brewster mirror (see Fig. 1). A superpolished gold mirror at incidence angle of $\sim$45$^\circ$ serves as a polarization analyzer predominantly reflecting the *s*-component of the XUV-radiation towards a micro-channel plate detector (MCP) with expected reflection ratio $|r_p|^2/|r_s|^2\approx$ 1:4-1:6 in the spectral range of 20–50 eV. The mirror and MCP-detector were mounted on a rotation stage and placed at the image plane of our XUV-spectrometer. This setup allows to measure the reflected XUV intensity as a function of the analyzer azimuthal angle $I(\alpha )$ in the polarization plane to obtain the polarimetric curve. Due to the axial symmetry of any polarization ellipse it is sufficient for the evaluation of the polarimetric curve to cover the range of $\alpha$ $\in$ [0;180] degrees. However this approximation could lead to faulty conclusions and erroneous values, since the symmetry of the polarization curve may be broken, if the rotation axis and the XUV-beam are not collinear. Thus, we scan the full 360 degrees range to provide a compliance for curve symmetry and validate the alignment. Another problem has to be solved is the inserting of a phase-retarder into the setup to conduct measurements for at least two settings (see Supplementary F for more details). The gold coated toroid and diffraction grating are acting as a phase retarder in our setup due to different reflection coefficients for *s*- and *p*- components and introduced phase difference. To get the necessary second retarder setting we have to rotate this retarder, that is technically challenging in our setup. Taking into account, that the essence of the second setting is to change the polarization state of the input beam in a very definitive way, we can profit from the inherent property of the TCBC approach, that the helicity of the harmonics follow the helicities of the driving fields. Under the same generation conditions, the exchange of the drivers’ helicities will only affect the helicity of the harmonic, and particularly leaves any unpolarized component unchanged. This action is tantamount to a pure $\pi$/2 phase variation of an ideal phase retarder approaching the RAE with compensator scheme. By conducting polarimetry measurements for both helicities, we can determine the Stokes vector by fitting Eq.  (1), which is derived for the setup on basis of the Stokes-Mueller formalism (for more details see Supplementary D), to the measured data for right-handed $+\tilde{S}_3$ and for left-handed $-\tilde{S}_3$ polarizations simultaneously.

1 $$\begin{aligned} I(\alpha ;\tilde{S}_1,\tilde{S}_2,\tilde{S}_3)&\propto 1 - \tilde{S}_1 \cos 2\psi _s-\cos 2\psi _a(\tilde{S}_1-\cos 2\psi _s)\,\cos 2\alpha \nonumber \\&+(\tilde{S}_2 \cos \Delta _s + \tilde{S}_3 \sin \Delta _s)\,\sin 2\psi _s \cos 2\psi _a\, \sin 2\alpha , \end{aligned}$$

where $\tilde{S}_i$ are the normalized Stokes parameters, $\psi _{s/a} = \arctan (|r_p|/|r_s|)_{s/a}$ the reflection parameters of the spectrometer and analyzer, and $\Delta$ is the phase-shift between $p-$ and $s-$component introduced by the spectrometer. The $\psi _{s/a}$ and $\Delta$ can be derived from tabulated quantities, however this requires exact knowledge of numerous experimental parameters, does not include possible individual surface degradation and will suffer on inaccuracy. To avoid a possible discrepancy we have determined these crucial parameters experimentally for individual harmonics (detailed description in Supplementary E). Table  1 gives an overview of the measured values.

**Table 1:** Parameters $|r_p|/|r_s| = \tan \psi$ and $\Delta _s$ for individual harmonic, where indices *a* and *s* denote the analyzer and spectrometer, respectively.

	$\tan (\psi _a)$	$\tan (\psi _s)$	$\Delta _s$, rad
H13	$0.49\pm 0.01$	$0.71\pm 0.01$	$2.64\pm 0.04$
H15	$0.44\pm 0.01$	$0.81\pm 0.01$	$2.59\pm 0.05$
H17	$0.38\pm 0.01$	$0.83\pm 0.01$	$2.69\pm 0.05$
H19	$0.42\pm 0.01$	$0.91\pm 0.01$	$2.67\pm 0.03$
H21	$0.39\pm 0.01$	$0.88\pm 0.01$	$2.66\pm 0.03$

For first verification we applied our approach to determine the Stokes vector of the elliptically polarized harmonics produced in the TCBC scheme under standard conditions, e.g. with $\sim$30 fs driving pulses, which have already been reported to be highly elliptical^31,43,46^. The spectral intensities of the generated harmonics stay unchanged upon interchanging the driver’s helicities. Moreover, the spectra demonstrate a high contrast to the forbidden harmonics with orders 3*n* (at least 10:1 of H16 to H15 and H18) indicating a high ellipticity of the harmonics. We determined the Stokes parameters for harmonic orders H13-H21 by fitting Eq. (1) simultaneously to the measured data, e.g. $I(\alpha ; \tilde{S}_1,\tilde{S}_2,+\tilde{S}_3))$ for right-handed and $I(\alpha ; \tilde{S}_1,\tilde{S}_2,-\tilde{S}_3))$ for left-handed polarization.

**Figure 3:**
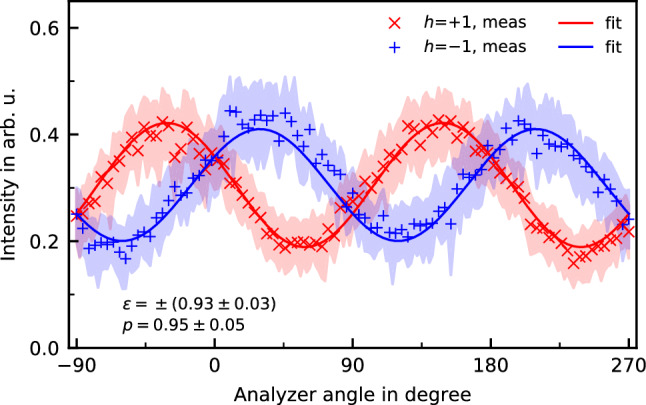
Measured intensities for harmonic order 16 in the cases of positive (blue, ‘+’) and negative helicity (red, ‘x’), with the shaded areas representing the standard deviation.

Figure 3 exemplarily shows results of the measurements and the corresponding fitting for harmonic H16. The fitting procedure (for details see Supplementary F) allows to determine the Stokes vector uniquely, yielding an ellipticity $|\epsilon |=0.93\pm 0.03$ and a degree of polarization $p = 0.95\pm 0.05$. By using the determined values for polarization degree and assigning helicity to the individual harmonics in accordance with the scheme of two-colour beams, we can modify the retrieving procedure for polarization state (see Supplementary D) Eq. (1) can be expressed in terms of $\chi$ as the ellipticity angle ($\epsilon = tan\chi$), $\theta$ as the ellipse orientation angle, and *p* as polarization degree:

2 $$\begin{aligned} I(\alpha ; C, \chi , \theta , p) =&C \cdot ( 1 - p\cos 2\chi \cos 2\theta \cos 2 \psi _s \nonumber \\&- \cos 2\psi _a(p\cos 2\chi \cos 2\theta -\cos 2\psi _s)\cos 2\alpha \nonumber \\&+ p(\cos 2\chi \sin 2\theta \cos \Delta + \sin 2\chi \sin \Delta ) \nonumber \\&\times \sin 2\psi _s \cos 2\psi _a \sin 2\alpha ), \end{aligned}$$

with *C* as proportionality constant.

With Eq. (2) we can achieve an unique result for the Stokes vector with a use of two polarimetric curve measured in RAE schema.

**Figure 4:**
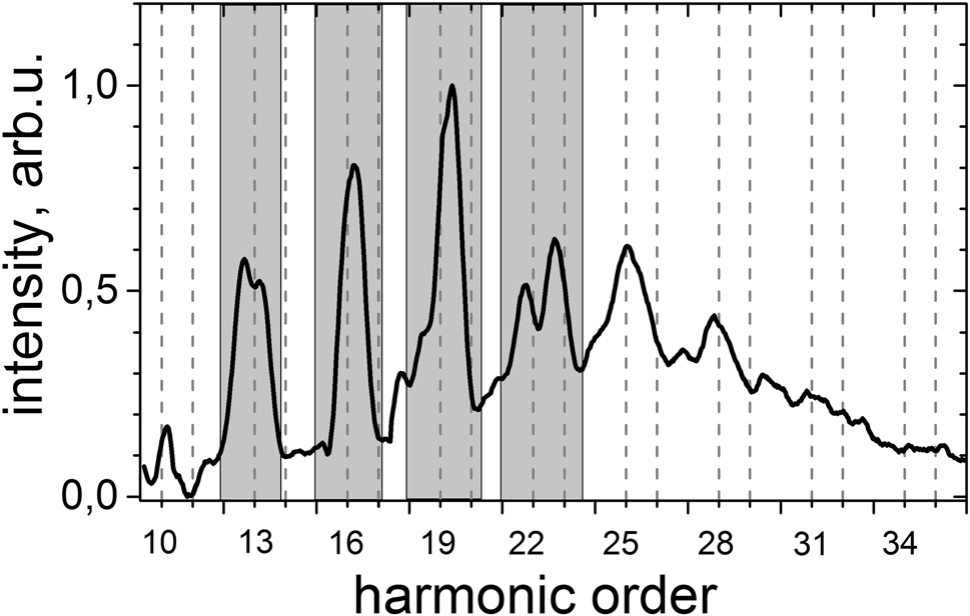
Shadowed area show parts of the XUV-continuum separated for polrization measurements.

The XUV-radiation generated with few-cycle driving pulses in Fig. 2 has a continuous spectral content dispersed at a large angle in the image plane of the spectrometer. To apply the developed polarimetric approach for characterization of the generated XUV-continuum, we have separated parts of the spectrum of about 3–4 eV width by a slit placed at the input of the rotating analyzer (Fig.  4).

**Figure 5:**
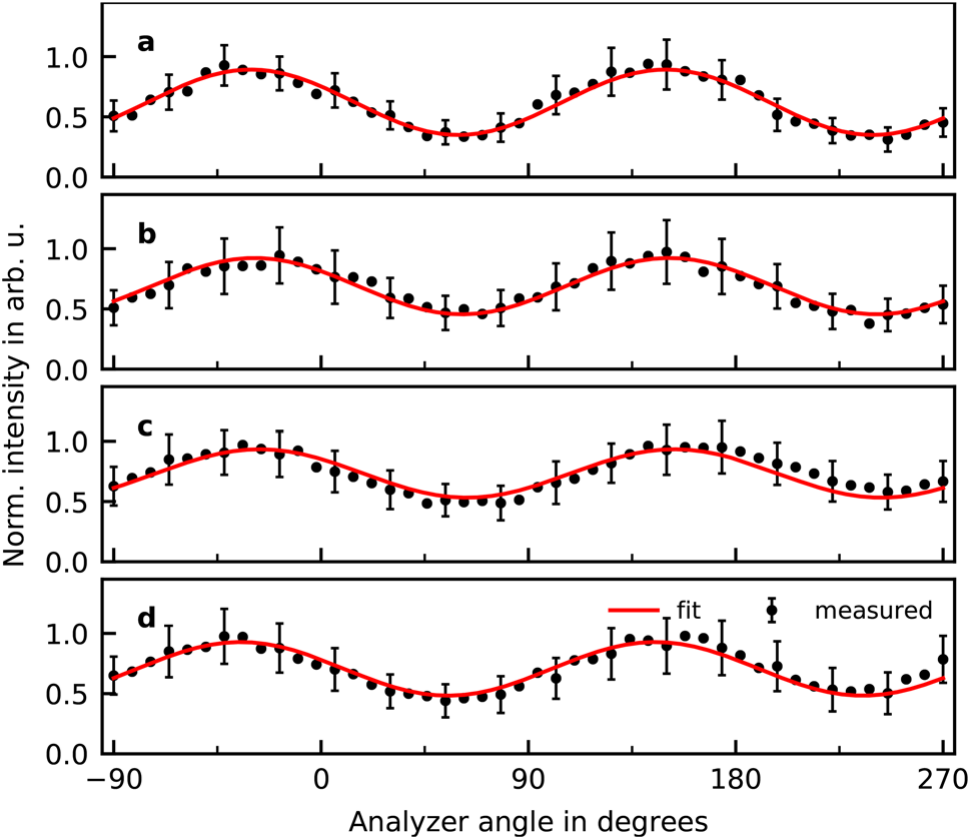
The recorded signal as a function of the analyzer azimuthal angle $\theta$ measuremed for XUV-radiation centered around harmonic with orders **(a)** H13, **(b)** H16, **(c)** H19 and **(d)** H22 from Fig. 2 for −1.8 fs delay.

The diffraction grating was adjusted to the several positions corresponding to the maxima observable in the spectrum to run data acquisition in robust and repeatable way. Figure 5 presents the corresponding polarimetry curves.

To evaluate data we apply the modified procedure by fitting Eq. (2) taking into account the previously determined polarization degree $p = 0.95\pm 0.05$ for our TCBC approach. The resulting parameters characterizing polarization states of harmonics are summarized in Table 2.

**Table 2:** Ellipticities $\epsilon$ and orientation angles $\theta$ retrieved by evaluating the polarimetry measurements for the harmonic radiation generated in the few-cycle setup at the position of the corresponding harmonics.

	$\epsilon = \tan \chi$	$\theta$
H13	$-0.80\pm 0.03$	$35.6^\circ \pm 4.6^\circ$
H16	$-0.91\pm 0.02$	$22.3^\circ \pm 2.0^\circ$
H19	$-0.82\pm 0.03$	$24.5^\circ \pm 3.0^\circ$
H22	$-0.94\pm 0.02$	$47.7^\circ \pm 1.8^\circ$

At this point we have determined the ellipticity of the most intense parts of the generated XUV radiation, however, to make a decisive conclusion about the polarization properties of the attosecond pulses including their entire spectral content, information about the continuous part of the spectrum in the cut-off region is substantial. Unfortunately, measurement of the spectral helicity in this spectral region is hampered by a drastic decrease in reflectivity (as for most common materials) of the polarizer mirror and increased intensity fluctuations typical for the cut-off region. As a result the measured data do not reach the necessary level of confidence to be included in the analysis. Further, we clearly define and separate results from measured polarization data and conclusions drawn solely from the spectral shape and arguments from the literature.

## Discussion

The ellipticity listed in Table 2 for high harmonics generated in our experiments have one of the highest reported to date. It is a remarkable result especially taking into account the relatively moderate ellipticity of the second harmonic driver of $\varepsilon$ = 0.88. A possible origin of such a low sensitivity in relation to the driver ellipticity, could lie in the intensity ratio $I(\omega )/I({2\omega })\approx$1:7 where the combined driving radiation is defined by the dominant fundamental circularly polarized beam and the contribution from the elliptical second harmonic beam is of minor importance. Harmonic generation at negative delays delivers another confirmation of this hypothesis. By this timing the second harmonic pulse overlaps with the satellite of the fundamental pulse setting the intensity ratio towards $I(\omega )/I({2\omega })<$1. In this position the second harmonic field plays a dominant role in the formation of the combined field, whose ellipticity gives rise to weak, but well perceivable 3*n* harmonics^31^. The low sensitivity towards imperfection of the circular polarization of one of the drivers presents a remarkable advantage of the TCBC approach over the noncollinear one-colour scheme, where the ellipticity of the generated XUV radiation depends drastically on the polarization of the driving fields and decreases sharply towards very moderate values of $\varepsilon _{XUV}$ $\approx$0.69 for even slightly elliptical driving fields with $\varepsilon _{(IR)}\approx$0.93^30^. Another advantage of the TCBC approach is a possibility to apply the driving radiation with a very broad range of the $I(\omega )$/$I(2{\omega })$ ratio (very different from requirement of equal intensities of two constituting beams in the noncollinear approach) performing spectral shaping of the generated XUV-spectrum^32,33,37^. The intensity ratio 7:1 used in our experiments fulfills a variety of crucial functions. First, the lower intensity of the second harmonic component results in a reduction of fast medium ionization, that in turn is responsible for decreasing harmonic emission within the driving envelope, associated with the depolarization effect^42^. Second, we almost eliminate the harmonics with orders $3n+2$, which are mainly affected by depolarizaton and possess lower and a fast varying ellipticity when the few-cycle pulses are applied^42^. Third, the forbidden harmonics of 3*n* orders remain strongly suppressed thanks to the major contribution to the driving field from the fundamental beam with circular polarization. The increasing contribution of the 3*n* harmonics results in a quick drop in harmonic ellipticity together with the corresponding growth of depolarized content and, therefore, its absence is an indirect evidence for generation of high elliptically polarized harmonics. Forth, the depolarization introduced by the variation of the relative $\omega -2\omega$ phase (e.g. if the CEP is not controlled) affects mostly harmonic orders $3n+2$, while maintaining the polarization degree as high as $p=0.93$^42^ for harmonics $3n+1$, that is close to the values of $p\approx 0.95$ determined in our measurements. The most drastic impact on the ellipticity of individual harmonics has its origin in the spectral overlap of harmonics with opposite helicity, i.e. the orders $3n+2$ and $3n+1$ when the width of harmonics extended as a consequence of very short duration of the driving fields, attosecond chirp on the rising edge of driving pulse envelope and inter-cycle temporal variation of harmonic dipole phase. We succeed to diminish this deterioration by utilising the helicity dependent filter and generate the highly spectral chiral XUV-continuum with the pronounced parts in the vicinity of $3n+1$ harmonics supporting generation of isolated attosecond pulses. The part of the continuum up to 35 eV corresponds to a transform-limited pulse of 190 as with less than 10% side-trailing pulses having ellipticity as high as $\varepsilon \ge 0.85$ (see Supplementary G). Despite of experimental multi-parameter optimisation the calculated ellipticity $\varepsilon \ge 0.85$ of the generated attosecond pulses differ from pure circular polarization. The main reason we suppose to be the effect of fast variation of driving pulse envelope. This variation breaks the symmetry of the three-fold driving laser field in amplitude and angular orientation followed by modification in phase and amplitude of the attosecond components and results in decreasing ellipticity. This presents a fundamental limitation for very short pulses having natural e.g. Gaussian envelopes. This limitation is also recognized by theoretical modeling, where the best envelope for driving pulse to produce the highest degree of ellipticity is defined as trapezoidal^42^.

Untill now we discuss the potential to generate isolated attosecond pulses based on the part of the generated XUV-spectrum, where the full Stokes vector was determined by polarimetry. Now we can summarize some very fundamental facts, which will support our hypothesis for high spectral chirality of the continuous part. Firstly, even though the spectrum is continuous, the peaks at positions of harmonics 3n + 1 are more intense compared to the rest of the radiation, i.e. H25, H28 and H32 in Fig. 4 a primarily specific feature for high spectral chirality. Secondly, numerical simulations^32,37,39^ also predict a homogeneous spectral helicity along the whole spectrum simmilar to the observable in our experiment with even increasing ellipticity in the region close to cut-off^32^. Moreover, the intensity ratio I(2w)/I(w) of 1:7 applied in our experiment is higher as used in the simulation (1:2.5), which likely increases the spectral chirality of the spectrum. Considering these results we can confidently assume the spectral and consequently polarization properties of the smooth continuum part above 38 eV to be similar as detected for the lower energetic part. Transform-limit for the entire generated spectrum (the details are presented in Supplementary G Figs. 6 and 7) demonstrate isolated 150 attosecond pulses. The ellipticity of the continuous part is presently a unknown value, but together with conclusions drawn from the literature we estimateit to be at least in a range of $\varepsilon \propto$[0:−0.77]. Taking into account the spectral amplitude we can approximate the resulting ellipticity of the pulses in a range of $\varepsilon \propto$[−0.5:−0.8].

In conclusion, we demonstrate the first successful implementation of the two-color bi-circular approach driven by few-cycle pulses to generate elliptically polarized broad spectrum spanning the range of 15–55 eV. By combining a 4.8 fs pulses and its second harmonic with 7.5 fs duration at the intensity ratio of 7:1 and utilizing optimal helicity dependent filter we are able to select harmonics with $(3n+1)$ orders and nearly completely suppress the harmonic of other orders. The clearly dominating harmonics are highly elliptical polarized with helicity of the fundamental component of the driving field and cause the resulting attosecond pulse train to follow this helicity. This conclusion drawn entirely from the spectral shape is further supported by the polarization characterization carried out by means of XUV-polarimetry. With measured ellipticity ranging from −0.8 to −0.94, the spectrum is highly elliptically polarized and its components all have the same helicity, offering ideal conditions for the generation of a highly elliptically polarized attosecond pulses. The generated XUV-continuum delivers a key prerequisite for generation of isolated pulses with a transform limited duration down to $\sim 150$ as. The ambitious task of direct determination of the temporal structure, phase and polarization state of attosecond pulses remains still a great challenge for experimental realization in the nearest future.

## Methods

### Experimental setup

Experiments have been performed with a Ti:sapphire-based laser system with a single stage regenerative amplifier producing 35 fs pulses with up to 1,5 mJ energy and central wavelength of $\sim$795 nm at 1 kHz repetition rate. The CEP of the pulses was not locked. The *p*-polarized laser beam was directed towards a hollow-core-fiber (HCF) for further pulse compression, followed by the the generation of *s*-polarized second harmonic beam in a 50 $\mu$m thick barium borate (BBO) crystal. The polarizations of fundamental and second harmonic beams were transformed to circular, but with opposite helicities by a superachromatic quarter-waveplate with spectral range of 310–1100 nm (for more details see Supplementary C). The dispersion control for both beams was applied separately resulting duration of 4.8 fs for the fundamental and of 7.5 fs for the second harmonic pulses.

## Supplementary Information

Supplementary Information.

## Data Availability

The data of this study are available from the corresponding author under reasonable request.
