# Supplementary material for: Elliptically polarized high-harmonic radiation for production of isolated attosecond pulses
Source: Sci Rep. 2021 May 5;11:9570. doi: 10.1038/s41598-021-88557-1 (PMC8099878; doi:10.1038/s41598-021-88557-1)
Supplement: Supplementary file 1 — Supplementary Information. [file 41598_2021_88557_MOESM1_ESM.pdf]

Supplementary information for
Elliptically polarized high-harmonic radiation for production of isolated attosecond pulses

Ulrich Bengs[1] and Nickolai Zhavoronkov[1*]

[1] Max-Born Institut Berlin, Max-Born Strasse 2A, 12489 Berlin, Germany and

[*] Corresponding author: zhavoron@mbi-berlin.de

I. SUPPLEMENTARY DOCUMENTS

A. Experimental and theoretical foundations for two-colour bi-circular approach

Two-colour bi-circular collinear scheme is a promising, intensively investigated approach for generation of circularly polarized harmonics, with well formulated fundamentals and operational principles [1–3]. Important, the exact spectral position and selection rules for the harmonic orders depend on spectral content of the driving radiation, where the frequency ratio of driving fields is decisive for specific selection rules [4]. In a case of a frequency ratio of 1:2 circularly polarized harmonics of orders $3n+1$ having the helicity as fundamental beam and of orders $3n+2$ following the helicity of the second harmonic beam are generated, while $3n$ harmonics are forbidden (n-being integer). To produce attosecond pulses of pure elliptical polarization, one group of harmonics has to be selected, via adjusting the intensity ratio of the components of the two-colour driving laser field [3, 5] accompanied by optimisation of phase-matching conditions for HHG in hollow fibers [5, 6] as well as in a thin gas cell [7]. In addition, the response from the atomic p-states is also helicity dependent and contributes to the suppression of $(3n+2)$ harmonic orders already at the single-atom level [3], taking advantage of nonzero magnetic quantum numbers $m \pm 1$ in the initial electronic state of Neon. Nowadays attosecond science routinely utilizes linear polarized pulses generated in forms of trains of attosecond pulses separated by half an optical cycle of the driving radiation. However, for many applications it is crucial to apply isolated attosecond pulses delivering experimental access to the fastest electron dynamics and enabling straightforward interpretation of spectroscopic data. Once the pulse train is generated, it is extremely difficult to isolate a single pulse, hence some time-gating process has to be applied already at generation events, such as spectral selection of half-cycle cutoffs [8–10], temporal polarization gating techniques [11, 12], and spatiotemporal gating via the attosecond lighthouse technique [13]. Such technologies are now relatively mature for application with linearly polarized harmonics, but in its infancy with regard to circularly polarized harmonics. Whereas the polarization gating approach, which utilizes time delayed counter-rotating laser beams, is universally applied for generation of linearly polarized isolated attosecond pulses, it can not be extended directly to application for elliptically polarized harmonics. Thus, the only reasonable route for pulse isolation lies in the use of few-cycle driving radiation.

Generation of isolated circularly polarized attosecond pulses still remains an important outstanding task. The key features of two-colour bi-circular collinear approach were addressed in numerous publications, among which Mediskauskas et. al. [14] provide the theoretical modeling for the generation of isolated attosecond pulses. The time-dependent Schrödinger equation (TDSE) was numerically solved for a neonlike model atom in the length gauge. The TDSE was propagated on a $2D$ Cartesian grid using a Taylor-series propagator with expansion up to eighth order. The sub-cycle dynamics of the emission process was analyzed using the Gabor transform of the time dependent acceleration dipoles. The driving field was combined from 800 nm and 400 nm counterrotating collinear laser beams with full width at half maximum duration of 4 fs, sin^2 envelope and $I = 1.7 \times 10^{14}\,W/cm^2$ peak intensity. It was shown, firstly, that circularly polarized attosecond pulses are generated already at the microscopic single atom level, thus formulating an essential difference to the noncollinear approach where local atom generates linearly polarized harmonics. Second, in the spectra obtained from adding the contributions from the $p+$ and $p-$ orbitals coherently, as required, harmonics corotating with the driving fundamental field dominate over those with opposite polarization. Third, the harmonic emission only occurs when both pulses overlap, and it was concluded, that TCBC approach could be naturally extended from the generation of an attosecond pulse train to the generation of an isolated attosecond pulse, using short driving pulses and utilizing a time delay between them. Other features important for the experimental implementation were disclosed in [15], where it was stated that the absolute value of the harmonic ellipticity generally increases with increasing harmonic order and that imperfection of the 2ω circular polarization is of larger influence on the ellipticity of $3n+2$ than on $3n+1$ orders especially for the lower harmonic orders. Barreau et.al. [16] have addressed the specific problem of depolarization and decreased ellipticity for the high harmonics generated with the TCBC approach. TDSE calculations in Helium and Argon show that harmonics can appear as only elliptical (not circular) already at the single atom level as a effect of short pulse envelope and fast ionization. Any breaking in symmetry between the XUV bursts within the fundamental cycle will result in a decreasing harmonic ellipticity and an increasing depolarization of individual harmonics. Such symmetry breaking results from fast temporal variation of the envelope at the rising/falling edges of a few-cycle pulse, manifesting a modification of the intensity and phase of emitted atto-bursts as compared to driving pulses with a slowly varying envelope ($\sim$100 fs). Short pulse envelope effect could

be intensified additionally by a variation of the carrier-envelope phase (CEP) of few-cycle pulses and are still significant for even 30 fs pulses. The presented modelling concludes, that the deterioration of the polarization state through depolarization and decreasing ellipticity mostly affects harmonics with orders $3n+2$ (in other notation $3n-1$) leaving the $3n+1$ harmonics less influenced [16]. This conclusion offers a good groundwork for the experimental realization of highly chiral harmonics via proper suppression of the harmonic orders $3n+2$ and most of the accompanying deteriorating effects.

B. Polarimetry of attosecond pulses

In general, attosecond pulse characterization is based on cross-correlation of harmonics beam with a laser field and relies on the measurement of photoionized electron spectrum (see e.g. overview [17]) to decode the attosecond spectral phase for complete reconstruction of the attosecond pulse. Such techniques are well developed for linearly polarized harmonics, but can not be directly transferred to harmonics with circular polarization because of the absence of cylindrical symmetry in the experimental implementation - the essential component for inversion algorithms. Very recently, Chen et. al. [18] introduced the tomographic reconstruction of circularly polarized high harmonics to provide a mean for the experimental determination of intensity and phase of the orthogonal fields components and to determine the ellipticity of the harmonics individually. In another approach the molecular frame photoelectron angular distributions in molecular dissociative photoionization is applied to probe the polarization state of ionizing radiation and encode the normalized Stokes parameters S_1, S_2 and S_3 (Stokes vector) describing the polarization state of individual harmonics [19]. Unfortunately these methods are extremely challenging in practical realization requiring very sophisticated experimental equipment, materials and data analysing procedures, including the atomic pure surface of samples, access to calibrated references measured on external XUV source, to electron-ion coincident $3D$ momentum spectroscopy and etc. The ellipticity of some specific harmonics in the vicinity of M-edges of the ferromagnetic 3d transition metals can be also determined with X-Ray magnetic circular dichroism (XMCD), however handling a typically very weak transmitted signal as well as the need of specific samples is extremely challenging [20]. Alternatively, the straight-forward approach of XUV-optical polarimetry [21] carried out with reflective optics and routinely used to analyse XUV radia-

tion at synchrotron and free electron-laser facilities [22], can find a reasonable application for high harmonics. The straight-forward approach of the rotating analyzer ellipsometry (RAE) is applied [1] to scan the azimuthal intensity distribution of the generated XUV radiation, in order to measure the polarization curve and to determine the polarization ellipse. However, since the S_3 parameter of Stokes vector is not accessible with this simple method, it lacks the ability to include a potential unpolarized light component, thereby providing only upper limit for ellipticity. The extended RAE approach resolves this issue by inserting a phase-retarder, denoted also as a compensator [21], into the setup and conducting measurements for at least two different retarder setting (see Supplementary I F). This method is in general much easier in realization and can be practically applied by integrating a polarimeter in existing XUV-spectrometer setups. The polarimetric scheme applied in [23] utilizes the type of phase retarder typically used at synchrotron facilities, faces a problem of low level signal, when harmonics experience reflections on numerous metallic surfaces. Full Stokes vector of non-collinear harmonics was retrieved afterward in iterative way with genetic algorithm including seven variables.

C. Generation of few-cycle driving pulses

Original output radiation from a single stage Ti:sapphire regenerative amplifier (Spitfire ACE) in the form of 35 fs pulses with central wavelength of ~795 nm and up to 1,5 mJ energy at 1 kHz repetition rate was coupled into a 1.2 meter long hollow-core-fiber (HCF) with inner diameter of 310 μm. The fiber was filled with Neon utilizing a gradient gas pressure along the HSF, where the pressure at the entrance of the fiber was kept around 1 mbar to avoid undesirable effects like buildup of plasma. The inlet gas pressure at the other end of the HCF is adjustable up to a maximum pressure of 3.5 bar. After nonlinear propagation in the HCF and subsequent phase-correction the pulses were characterized by using SPIDER (spectral interferometry for direct electric field reconstruction) revealing the pulses with duration of 3,6 fs and energy of 1 mJ. Figure 1 presents the corresponding temporal and spectral profiles.

The output from the HCF compressor was directed towards 50 μm thin beta-barium-borate crystal (BBO) to generate second harmonic pulses with about 65 μJ energy. The fundamental and the converted beams passed through the separate dispersion controls to

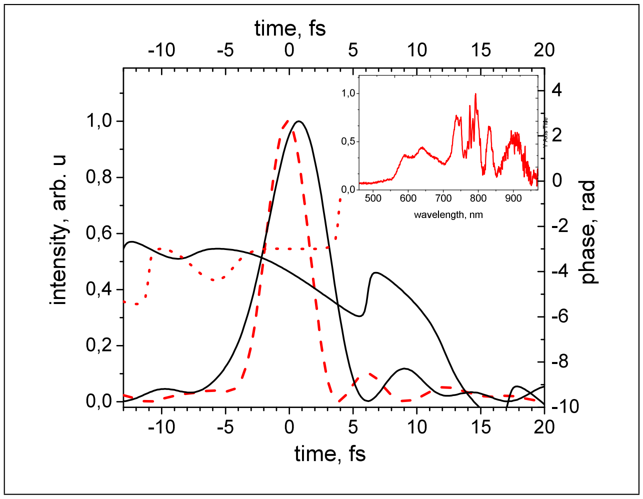

FIG. 1. The fundamental driving pulse directly after HCF together with spectrum (insert) and spectral phase (red curve) and after compensation of dispersion from optical elements at the position of target (black curves).

compensate additional dispersion from the optical elements on the path to the target. A piezo-driven delay stage controls delay between the two beams with attosecond accuracy, where zero delay was determined as maximum of cross-correlation function by sum-frequency mixing in nonlinear crystal. The p-polarized fundamental and s-polarized second harmonic beam was recombined in accurate collinear configuration and directed towards a super-achromatic quoter-wave-plate where the linear polarizations of these beams were converted simultaneously to circular polarizations, resulting the fundamental beam with clockwise circular polarization $\epsilon_{\text{red}} \approx 0.95$ and counter-rotating second harmonic beam $\epsilon_{\text{blue}} \approx 0.88$. The helicity of the beams could be changed to the opposite ones by corresponding reorientation of the superachromatic quoter-wave-plate. The pulses are temporally characterized using SPIDER for the fundamental and the self-diffraction frequency resolved gating (SD-FROG) for the second harmonic, with results shown in Figs. 1 and 2for 4,8 fs fundamental and 7.5 fs second harmonic pulses.

The two-colour driving radiation was focused into the 0.9 mm thick Ne-cell target, yielding the intensities at the focus of $I(w) \cong 7 \times 10^{14}\ W/cm^2$ and $I(2w) \cong 1 \times 10^{14}\ W/cm^2$. The emitted high harmonics were analysed by an HHG imaging spectrometer in 2f-2f configuration consisting of the gold coated 1 meter toroidal mirror and plane 300 line/mm grating at grazing incidence angle. The spectrally resolved XUV radiation was detected by microchan-

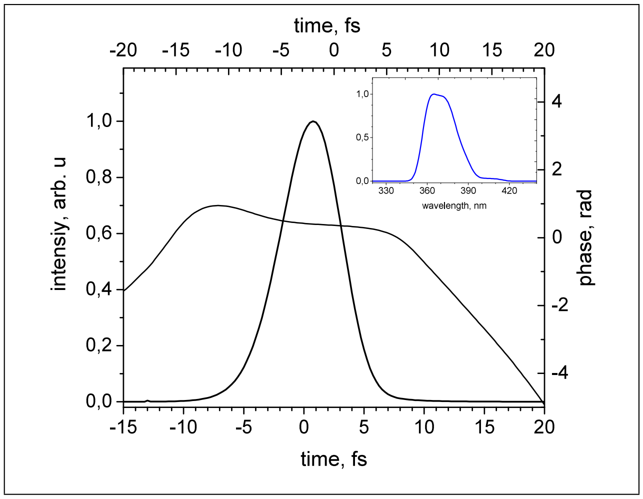

FIG. 2. The second harmonic driving pulse measured at the position of target (black curves) with the corresponding spectrum (insert).

nel plate (MCP) based XUV-detector with a phosphor screen and recorded by fast CMOS camera (PointGrey).

D. Stokes-Mueller Formalism

The polarization of light is completely characterized by the four Stokes parameters $\vec{S} = (S_0,\, S_1,\, S_2,\, S_3)$, that are defined by the relations

$$\begin{aligned} S_0 &= I_x + I_y \\ S_1 &= I_x - I_y \\ S_2 &= I_{+45^\circ} - I_{-45^\circ} \\ S_3 &= I_R - I_L, \end{aligned} \tag{1}$$

where I_x, I_y and $I_{\pm 45^\circ}$ are the intensities of light measured after a perfect polarizer oriented along $x-$, $y-$ and $\pm 45^\circ$ direction and $I_{\mathrm{R/L}}$ are the intensities measured along $\pm 45^\circ$ direction after a retardation of $\pi/2$ is applied. Note, that throughout this work the light polarized along the x-direction corresponds to p-polarization and light polarized along the y-direction corresponds to s-polarization. For convenience, we will use the normalized Stokes parameters $\tilde{S}_i = S_i/S_0, (i = 1, 2, 3)$. Moreover, the stokes parameters allow to describe partially

polarized light, with the degree of polarization

$$p = \sqrt{\tilde{S}_1^2 + \tilde{S}_2^2 + \tilde{S}_3^2} \leq 1, \tag{2}$$

where $p = 1$ for fully polarized light. It is also useful to express the polarization as an ellipse with orientation angle θ and ellipticity angle χ (ellipticity $\epsilon = \tan\chi$), so that the Stokes vector reads

$$\vec{S} = \begin{pmatrix} 1 \\ p\cos 2\chi \cos 2\theta \\ p\cos 2\chi \sin 2\theta \\ p\sin 2\chi \end{pmatrix}. \tag{3}$$

From the definition in Eq. (3) one can derive the relations for the ellipticity and orientation angle

$$\epsilon = \tan\left(\frac{1}{2}\arctan\frac{S_3}{\sqrt{S_1^2 + S_2^2}}\right) \tag{4}$$

$$\theta = \frac{1}{2}\arctan\frac{S_2}{S_1}. \tag{5}$$

Optical elements may change the polarization of light due to different reflectivities along its $p-$ and $s-$axis or by an induced phase-shift between these components upon reflection. Mathematically, this is described by the multiplication of a 4×4 Mueller-matrix, which in its most general form is represented by:

$$M_{\psi,\Delta} = A\begin{pmatrix} 1 & -\cos 2\psi & 0 & 0 \\ -\cos 2\psi & 1 & 0 & 0 \\ 0 & 0 & \sin 2\psi\cos\Delta & \sin 2\psi \sin\Delta \\ 0 & 0 & -\sin 2\psi\sin\Delta & \sin 2\psi\cos\Delta \end{pmatrix}, \tag{6}$$

where $\psi = \arctan(|r_p|/|r_s|)$ incorporates the ratio of reflection coefficients for light polarized along p- and s-direction and Δ is the introduced phase shift between p- and s-polarized components upon reflection on the optical element. Since we are not dealing with absolute intensities in this work, we will drop the factor A from here on. The Stokes vector after passing the setup described in section (?) then reads

$$\vec{S}_{\text{out}} = R(\alpha)M_{\psi a,\Delta a}R(-\alpha)M_{\psi s,\Delta s}\vec{S}_{\text{in}}, \tag{7}$$

where $M_{\psi s,\Delta}$ describes the spectrometer components and $R(\alpha)M_{\psi a,\Delta a}R(-\alpha)$ describes the rotating analyzer with the rotation matrix

$$R(\alpha) = \begin{pmatrix} 1 & 0 & 0 & 0 \\ 0 & \cos 2\alpha & \sin 2\alpha & 0 \\ 0 & -\sin 2\alpha & \cos 2\alpha & 0 \\ 0 & 0 & 0 & 1 \end{pmatrix}. \tag{8}$$

Since the detector used in this work is not phase sensitive, we can set the phase shift induced by the analyzer $\Delta_a = 0$. Also, while it is necessary to transform into the analyzer frame via $R(-\alpha)$, we can drop $R(\alpha)$ because of the isotropy of the detector. The detector measures the total intensity of the light I, which is by definition the first parameter of $\vec{S}_{\text{out}}$, yielding

$$\begin{aligned} I(\alpha; \tilde{S}_1, \tilde{S}_2, \tilde{S}_3) \propto 1 - \tilde{S}_1 \cos 2\psi_s - \cos 2\psi_a(\tilde{S}_1 - \cos 2\psi_s) \cos 2\alpha \\ + (\tilde{S}_2 \cos \Delta_s + \tilde{S}_3 \sin \Delta_s) \sin 2\psi_s \cos 2\psi_a \sin 2\alpha. \end{aligned} \tag{9}$$

Inserting Eq,3 into Eq.9 yields:

$$\begin{aligned} I(\alpha; C, \chi, \theta, p) =& C \cdot (1 - p \cos 2\chi \cos 2\theta \cos 2\psi_s \\ & - \cos 2\psi_a(p \cos 2\chi \cos 2\theta - \cos 2\psi_s) \cos 2\alpha \\ & + p(\cos 2\chi \sin 2\theta \cos \Delta + \sin 2\chi \sin \Delta) \\ & \times \sin 2\psi_s \cos 2\psi_a \sin 2\alpha), \end{aligned} \tag{10}$$

where C is the proportionality constant.

E. Measurement of system parameters

Before being able to measure the Stokes parameters it is necessary to determine the system parameters ψ_s, ψ_a and Δ_s. In principle, they can be derived from the reflection coefficients of gold which are available as tabulated quantities, however they critically depend on the angle of incidence of the light on the respective surfaces, which is difficult determine experimentally and moreover, on optical degradation of the reflecting surfaces, such as carbon contamination, that might cause a significant deviation from tabulated values. Therefore we determine these parameters experimentally by using a source of very

well known polarization, e.g. HHG driven by a linearly polarized mid-IR laser (800 nm, 35 fs), where the polarization of the driving laser is imprinted on the harmonics and can thoroughly be controlled using a half-wave plate. We record the intensity of every single harmonic as a function of the analyzer angle for three different linear polarization angles of the high-harmonic radiation: p- and s-polarized light ($\vec{S}_{\mathrm{p/s}} = (1, \pm 1, 0, 0)$) and polarization angles 45° ($\vec{S}_{45^\circ} = (1, 0, 1, 0)$). In these cases Eq. (9) simplifies to:

$$I(\alpha; \pm 1, 0, 0) \propto 1 \mp \cos 2\psi_s - \cos 2\psi_a(\pm 1 - \cos 2\psi_s)\, \cos 2\alpha \tag{11}$$

$$\begin{aligned} I(\alpha; 0, +1, 0) \propto\ & 1 + \cos 2\psi_a \cos 2\psi_s \cos 2\alpha \\ & + \cos \Delta_s \sin 2\psi_s \cos 2\psi_a\, \sin 2\alpha. \end{aligned} \tag{12}$$

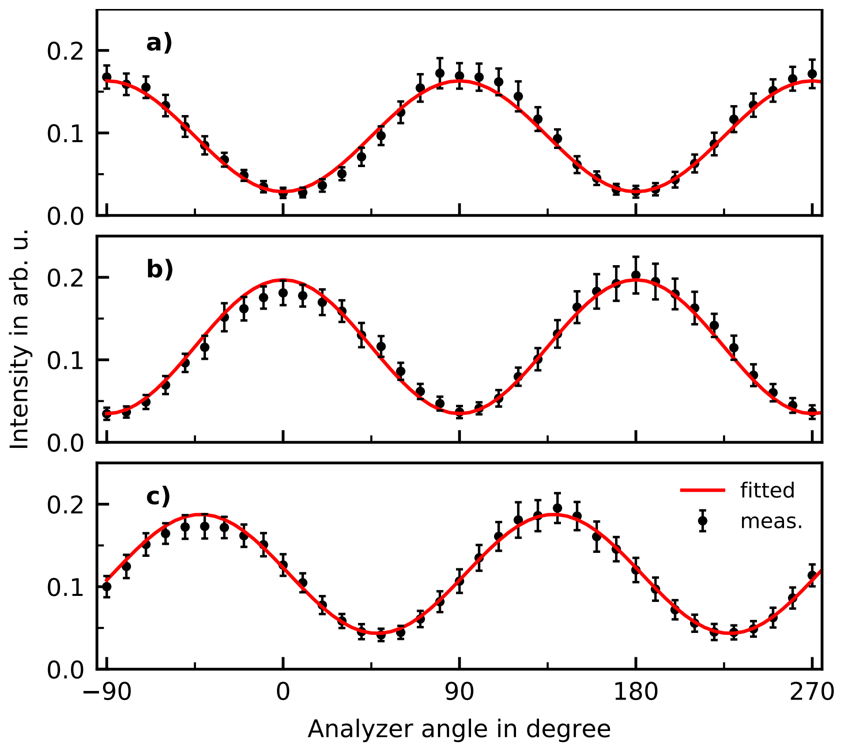

FIG. 3. Measured XUV-intensity (black) as a function of the analyzer azimuthal angle α, exemplary presentation for harmonic order 19 for **a)** p-polarization, **b)** s-polarization and **c)** 45°-polarization. The red line corresponds to the best fit to the data.

In the first step, we conduct polarimetry measurements for *s*- and *p*-polarized light and since there is no dependence of Δ_s we obtain the parameters ψ_a and ψ_s by fitting Eq. (**??**). In the second step, we use light polarized along 45° and fit Eq. (12) with Δ_s as the only unknown parameter to the data. ψ_a, ψ_s and Δ_s are frequency dependent and therefore need

to be determined for each photon energy of interest. Fig. 3 shows the measured data (black) and the fitted curves (red) for harmonic 19, where each data point represents an average over ∼120 measurements and the error bars correspond to standard deviation. Table ?? shows the obtained setup parameters for each individual harmonic.

F. Complete measurement of all Stokes-parameters

The measured by our analyzer (RAE) polarization curve can be in general expressed by the trigonometric function $I(\alpha)$ in the form:

$$I(\alpha) = a + b\cos 2\alpha + c\sin 2\alpha, \tag{13}$$

with three real parameters a, b and c that can be retrieved independently by fitting Eq.13 to data measured in the experiment. These parameters are proportional to constant C, the ellipticity angle χ and the orientation angle θ in Stokes formalism Eq.10. However, there is fourth free parameter that need to be determined to retrieve the full Stokes vector (S_1, S_2, S_3 and a proportionality constant) - the degree of polarization p. In order to find a unique solution for a single measured curve, one has to be assign a concrete value to one of the parameters, i.e. p, and to determine the other parameters by fitting. This problem is illustrated in Fig. 4 where red curves are equal, but correspond to the different combinations of unpolarized and elliptically polarized light. To solve this problem in general, we have to add an additional degree of freedom for p to Eq.13, that is solved by including a rotatable phase retarder (often called *compensator*) into the setup and perform polarimetry for different compensator settings with the well defined phase shifts. The full Stokes vector can be measured in RAE with compensator and the degree of polarization p can also be determined from this technique. The unique solution can then be found by measuring at least two polarization curves. However, the space limitation in the current setup does not allow to insert a XUV-compensator, which often comes in the form of a stack of three or more mirrors. Another negative aspect is drastic drop of the transmitted radiation due to multiple partial reflections on mirrors.

Here we propose an alternative technique, which exploits the polarization properties dictated by the symmetry of the driving fields in TCBC approach for chiral HHG: harmonic orders $(3n + 1)$ have the same helicity as the fundamental driving field and orders $(3n + 2)$ inherit

the helicity of the second harmonic. Thus, the helicity of each harmonic could be changed to opposite one by interchanging the helicity of the driving fields. It is very legitimate to assume, that under equal generation conditions for both helicities, the change of helicity's sign will only affect the sign of the Stokes parameter S_3. The intensity at the detector then reads

$$\begin{aligned} I_{\pm}(\alpha; \tilde{S}_1, \tilde{S}_2, \pm\tilde{S}_3) \propto\, & 1 - \tilde{S}_1 \cos 2\psi_s \\ & - \cos 2\psi_a (\tilde{S}_1 - \cos 2\psi_s)\, \cos 2\alpha \\ & + (\tilde{S}_2 \cos \Delta_s \pm \tilde{S}_3 \sin \Delta_s)\, \sin 2\psi_s \cos 2\psi_a\, \sin 2\alpha, \end{aligned} \tag{14}$$

where the sign of S_3 depends on the helicity of the particular harmonic. I_+ and I_- are linearly independent, so that the combination of the two equations allows us to include the last remaining parameter to the fitting procedure. Therefore, we are able to determine all Stokes parameters from two measurements by performing polarimetry for a single harmonic with both possible helicities and fitting I_+ and I_- simultaneously to both data sets (i.e. with shared Stokes parameters).

Fig. 4 illustrates different outcome scenarios for this type of measurement, where the intensity is plotted as a function of the azimuthal angle α. The red curves in all panels **a)-d)** are identical, however they were created using different sets of Stokes parameters, i.e. with different degrees of polarization and ellipticity. Without any additional information it is impossible to uniquely determine the state of polarization from this. However, conducting a second measurement (blue curves), where only the helicity of the harmonic is changed resolves this issue. For $p = 1$ the minimum to maximum ratio of the intensities is identical for both helicities. The lower the degree of polarization, the higher is the discrepancy in the minimum to maximum ratio between both helicities.

G. Temporal envelopes of XUV pulses

The important question related to a temporal structure of the generated radiation can be clarified by calculating transform-limited pulses of the measured HHG spectrum or its parts. Results in Fig. 5 and Fig. 6 provide an estimate of the temporal shape of the radiation and the shortest possible duration of the generated attosecond pulses by selecting different parts of the generated spectrum. Notwithstanding which part of the spectrum is picked out the

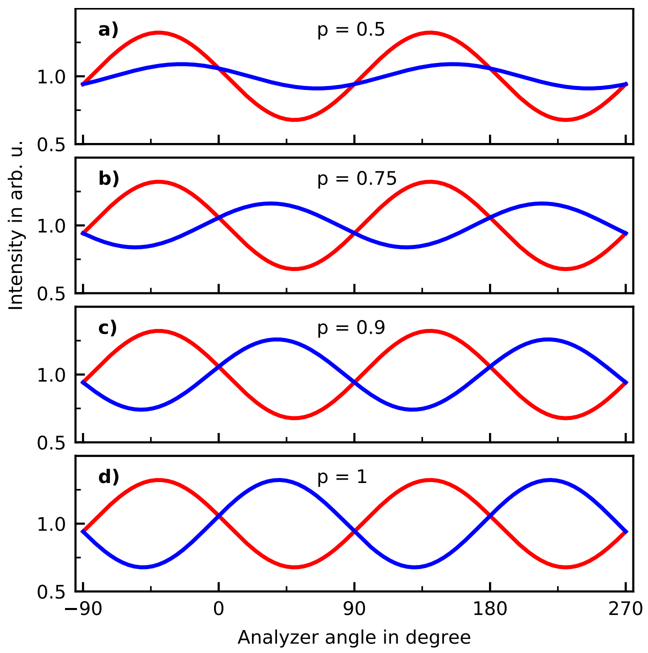

FIG. 4. Measuring the polarization of light with an analyzer alone is not sufficient to determine all Stokes-parameters independently, e.g. a measured curve can correspond to perfectly circularly polarized light or a combination of elliptically polarized and unpolarized light. The red curves correspond to different combination of polarized and unpolarized light with **a)** $p = 0.5$, $\varepsilon = 0.48$ ($\vec{S} = (1, 0, 0.31, -0.39)$), **b)** $p = 0.75$, $\varepsilon = 0.82$ ($\vec{S} = (1, 0, 0.13, -0.74)$), **c)** $p = 0.9$, $\varepsilon = 0.95$ ($\vec{S} = (1, 0, 0.05, -0.9)$) and **d)** $p = 1$, $\varepsilon = 1$ ($\vec{S} = (1, 0, 0, -1)$). The blue lines correspond to light with the same Stokes parameters except for the change of sign in S_3.

temporal structures present an isolated attosecond pulse. Even for the mostly modulated low energetic part of the spectrum within H13-H22, where we experimentally confirmed high ellipticity, temporal structure in Fig. 5 and in Fig. 7 show isolated pulse with very weak satellites do not exceeding 10% of main pulse intensity. Filtering the energetically highest part of the spectrum in the cut-off region supports a true isolated pulse with the intensity profile shown in Fig. 6. The pulses with duration as short as 145 as can be produced. However in practice generation of transform-limited pulses usually is hindered by inherent for HHG process nonlinear phase prolonging the duration towards 250-300 as, fortunately, without affecting isolated structure of the attossecond pulses.

Additionally considering the measured polarization of H13-H22 in the transform-limited pulse yields an overall ellipticity of $\epsilon \approx 0.87$.

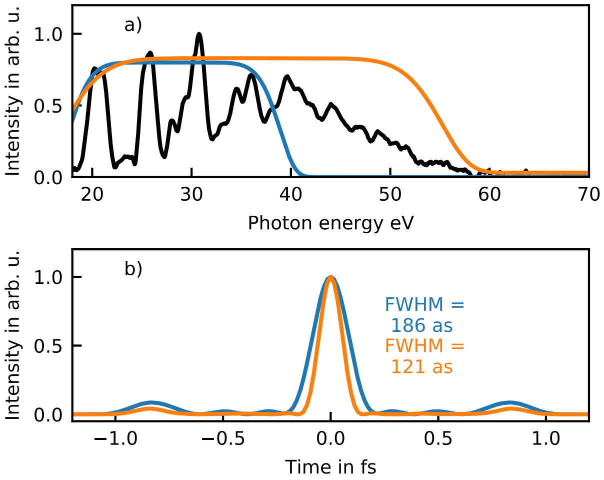

FIG. 5. a) Measured spectrum (black) and band-pass filters (orange, blue) to select different spectral regions. b) Corresponding intensity profiles in time-domain.

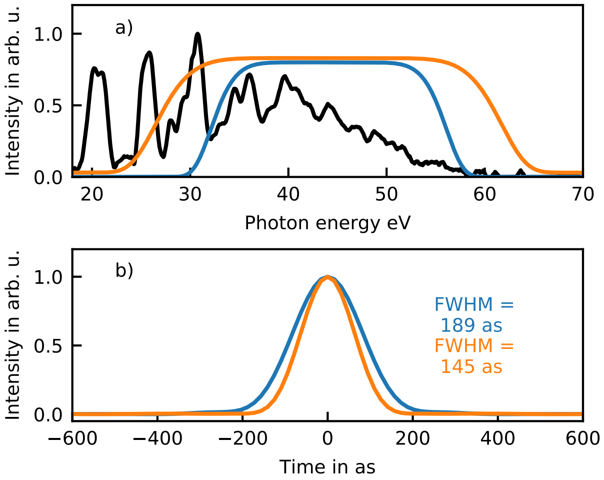

FIG. 6. a) Measured spectrum (black) and band-pass filters (orange, blue) to select the continuum part of the spectrum. b) Corresponding intensity profiles in time-domain.

Fig. 7 shows the transverse electric field of this pulse in time domain. We assume only a minor contribution from counter-rotating harmonics due to their weak intensities and have therefore neglected them in this evaluation.

[]

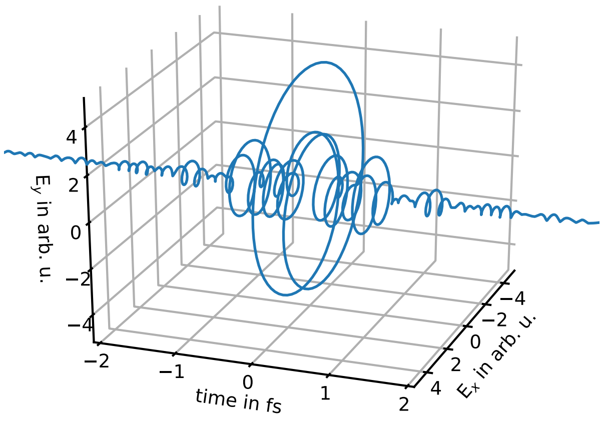

FIG. 7. Electric field of the transform-limited pulse with the spectral content of 20-34 eV (H13-H22), where high ellipticity was determined experimentally.

II. REFERENCES

[1] A. Fleischer, P. Sidorenko, and O. Cohen, Opt. Lett., OL **38**, 223 (2013).

[2] T. Fan, P. Grychtol, R. Knut, C. Hernández-García, D. D. Hickstein, D. Zusin, C. Gentry, F. J. Dollar, C. A. Mancuso, C. W. Hogle, O. Kfir, D. Legut, K. Carva, J. L. Ellis, K. M. Dorney, C. Chen, O. G. Shpyrko, E. E. Fullerton, O. Cohen, P. M. Oppeneer, D. B. Milošević, A. Becker, A. A. Jaroń-Becker, T. Popmintchev, M. M. Murnane, and H. C. Kapteyn, Proceedings of the National Academy of Sciences of the United States of America **112**, 14206 (2015).

[3] A. Jiménez-Galán, N. Zhavoronkov, D. Ayuso, F. Morales, S. Patchkovskii, M. Schloz, E. Pisanty, O. Smirnova, and M. Ivanov, Physical Review A **97**, 023409 (2018).

[4] D. B. Milošević, Phys. Rev. A **98**, 033405 (2018).

[5] K. M. Dorney, J. L. Ellis, C. Hernández-García, D. D. Hickstein, C. A. Mancuso, N. Brooks, T. Fan, G. Fan, D. Zusin, C. Gentry, P. Grychtol, H. C. Kapteyn, and M. M. Murnane, Physical Review Letters **119**, 063201 (2017).

[6] O. Kfir, P. Grychtol, E. Turgut, R. Knut, D. Zusin, A. Fleischer, Eliyahu Bordo, T. Fan, D. Popmintchev, T. Popmintchev, H. Kapteyn, M. Murnane, and O. Cohen, Journal of

Physics B: Atomic, Molecular and Optical Physics **49**, 123501 (2016).

[7] N. Zhavoronkov and M. Ivanov, Opt. Lett., OL **42**, 4720 (2017).

[8] A. Baltuška, T. Udem, M. Uiberacker, M. Hentschel, E. Goulielmakis, C. Gohle, R. Holzwarth, V. S. Yakovlev, A. Scrinzi, T. W. Hänsch, and F. Krausz, Nature **421**, 611 (2003).

[9] E. Goulielmakis, M. Schultze, M. Hofstetter, V. S. Yakovlev, J. Gagnon, M. Uiberacker, A. L. Aquila, E. M. Gullikson, D. T. Attwood, R. Kienberger, F. Krausz, and U. Kleineberg, Science **320**, 1614 (2008).

[10] A. Jullien, T. Pfeifer, M. J. Abel, P. M. Nagel, M. J. Bell, D. M. Neumark, and S. R. Leone, Appl. Phys. B **93**, 433 (2008).

[11] G. Sansone, E. Benedetti, F. Calegari, C. Vozzi, L. Avaldi, R. Flammini, L. Poletto, P. Villoresi, C. Altucci, R. Velotta, S. Stagira, S. D. Silvestri, and M. Nisoli, Science **314**, 443 (2006).

[12] H. Mashiko, S. Gilbertson, C. Li, S. D. Khan, M. M. Shakya, E. Moon, and Z. Chang, Phys. Rev. Lett. **100**, 103906 (2008).

[13] H. Vincenti and F. Quéré, Phys. Rev. Lett. **108**, 113904 (2012).

[14] L. Medišauskas, J. Wragg, H. van der Hart, and M. Y. Ivanov, Phys. Rev. Lett. **115**, 153001 (2015).

[15] D. B. Milošević, W. Becker, and R. Kopold, Physical Review A **61**, 063403 (2000).

[16] L. Barreau, K. Veyrinas, V. Gruson, S. J. Weber, T. Auguste, J.-F. Hergott, F. Lepetit, B. Carré, J.-C. Houver, D. Dowek, and P. Salières, Nature Communications **9**, 4727 (2018).

[17] Z. Chang and P. Corkum, J. Opt. Soc. Am. B, JOSAB **27**, B9 (2010).

[18] C. Chen, Z. Tao, C. Hernández-García, P. Matyba, A. Carr, R. Knut, O. Kfir, D. Zusin, C. Gentry, P. Grychtol, O. Cohen, L. Plaja, A. Becker, A. Jaron-Becker, H. Kapteyn, and M. Murnane, Science Advances **2**, e1501333 (2016).

[19] K. Veyrinas, V. Gruson, S. J. Weber, L. Barreau, T. Ruchon, J.-F. Hergott, J.-C. Houver, R. R. Lucchese, P. Salières, and D. Dowek, Faraday Discuss. **194**, 161 (2016).

[20] F. Willems, C. T. L. Smeenk, N. Zhavoronkov, O. Kornilov, I. Radu, M. Schmidbauer, M. Hanke, C. von Korff Schmising, M. J. J. Vrakking, and S. Eisebitt, Physical Review B **92**, 220405 (2015).

[21] H. Fujiwara, *Spectroscopic Ellipsometry: Principles and Applications* (John Wiley & Sons, 2007).

[22] C. von Korff Schmising, D. Weder, T. Noll, B. Pfau, M. Hennecke, C. Strüber, I. Radu, M. Schneider, S. Staeck, C. M. Günther, J. Lüning, A. e. d. Merhe, J. Buck, G. Hartmann, J. Viefhaus, R. Treusch, and S. Eisebitt, Review of Scientific Instruments **88**, 053903 (2017).

[23] P.-C. Huang, C. Hernández-García, J.-T. Huang, P.-Y. Huang, C.-H. Lu, L. Rego, D. D. Hickstein, J. L. Ellis, A. Jaron-Becker, A. Becker, S.-D. Yang, C. G. Durfee, L. Plaja, H. C. Kapteyn, M. M. Murnane, A. H. Kung, and M.-C. Chen, Nature Photonics **12**, 349 (2018).
